# Loss of Ca^2+^ entry via Orai–TRPC1 induces ER stress, initiating immune activation in macrophages

**DOI:** 10.1242/jcs.237610

**Published:** 2019-12-18

**Authors:** Viviane Nascimento Da Conceicao, Yuyang Sun, Emily K. Zboril, Jorge J. De la Chapa, Brij B. Singh

**Affiliations:** 1 Department of Periodontics, School of Dentistry, University of Texas Health San Antonio, San Antonio, TX 78229, USA; 2 Department of Comprehensive Dentistry, School of Dentistry, University of Texas Health San Antonio, San Antonio, TX 78229, USA

**Keywords:** SOCE channels, Ca^2+^ modulation, TRPC1, ER stress, Immune activation

## Abstract

Activation of cellular stresses is associated with inflammation; however, the mechanisms are not well identified. Here, we provide evidence that loss of Ca^2+^ influx induces endoplasmic reticulum (ER) stress in primary macrophages and in murine macrophage cell line Raw 264.7, in which the unfolded protein response is initiated to modulate cytokine production, thereby activating the immune response. Stressors that initiate the ER stress response block store-dependent Ca^2+^ entry in macrophages prior to the activation of the unfolded protein response. The endogenous Ca^2+^ entry channel is dependent on the Orai1–TRPC1–STIM1 complex, and the presence of ER stressors decreased expression of TRPC1, Orai1 and STIM1. Additionally, blocking Ca^2+^ entry with SKF96365 also induced ER stress, promoted cytokine production, activation of autophagy, increased caspase activation and induced apoptosis. Furthermore, ER stress inducers inhibited cell cycle progression, promoted the inflammatory M1 phenotype, and increased phagocytosis. Mechanistically, restoration of Orai1–STIM1 expression inhibited the ER stress-mediated loss of Ca^2+^ entry that prevents ER stress and inhibits cytokine production, and thus induced cell survival. These results suggest an unequivocal role of Ca^2+^ entry in modulating ER stress and in the induction of inflammation.

## Teaser

**Summary:** Channel proteins TRPC1 and Orai1 modulate Ca^2+^ entry, which inhibits ER-stress induced activation of macrophages and the development of the M1 phenotype.

## INTRODUCTION

The endoplasmic reticulum (ER) is a vital organelle responsible for various cellular functions, such as lipid and protein synthesis and regulation of Ca^2+^ homeostasis (Braakman and Hebert, 2013; Parekh and Putney, 2005). Importantly, cellular stress provokes the release of pro-inflammatory cytokines that stimulate the inflammatory response (Liu et al., 2017; Pinton et al., 2008); however, the mechanisms by which ER stress leads to immune activation are not well known. In non-excitable cells, change in intracellular Ca^2+^ ([Ca^2+^]_i_) levels directly related to the release of Ca^2+^ stores from the ER, is due to activation of the store-operated Ca^2+^ entry (SOCE) mechanism (Tojyo et al., 2014). Once ER Ca^2+^ stores are depleted, stromal interaction molecule 1 (STIM1), which acts as an ER sensor, interacts with proteins at the plasma membrane Ca^2+^ influx channels (Liao et al., 2008; Shaw and Feske, 2012a).

SOCE in immune cells is mediated by the highly Ca^2+^-selective Ca^2+^ release-activated Ca^2+^ (CRAC) channel and is essential for the proper immune response (Shaw and Feske, 2012a; Vaeth et al., 2015). An increase in [Ca^2+^]_i_ is initiated by agonist binding to cell-surface receptors, which generates second messenger IP_3_, leading to the release of Ca^2+^ stored in the ER. Release of ER Ca^2+^ allows ER protein STIM1 to aggregate, and also to interact with Orai1 (Feske et al., 2006; Liao et al., 2008) and transient receptor potential canonical 1 (TRPC1) channels (Asanov et al., 2015; Liao et al., 2008), thus increasing the [Ca^2+^]_i_ that is essential for cellular functions (Parekh and Putney, 2005; Putney et al., 2017). Importantly, immune cell activation is also dependent on SOCE, and loss of Orai1 or STIM1 has been shown to lead to an impaired immune function (Shaw and Feske, 2012b). Another mechanism that also affects the innate and adaptive immune response is ER stress, with chronic ER stress contributing to abnormal physiological processes involved in disease pathogenesis and progression (Islam et al., 2006; Kaufman, 1999; Kim et al., 2013; Todd et al., 2008). Moreover, abnormal immune activation caused by chronic ER stress is also associated with autoimmune and inflammatory disorders such as diabetes, atherosclerosis, myositis and inflammatory bowel disease (Arruda and Hotamisligil, 2015). Thus, establishing the mechanism(s) by which ER stress induces immune activation is essential for research into potential treatments for these diseases.

The ER not only plays an important role in Ca^2+^ signaling but is also essential for protein synthesis and proper protein folding and modification (Braakman and Hebert, 2013). More than half of newly synthesized proteins translocate to the lumen of the ER, for folding and targeting to various cellular organelles or to be transported to the surface of the cell. (Görlach et al., 2006; Kaufman, 1999; Lawless and Greene, 2012; Zhang and Kaufman, 2008). Ca^2+^ is required for the chaperone activity of many ER proteins, and loss of this vital function induces ER stress. In addition, genetic or chemical inhibition can also activate the ER stress response, through a common signaling mechanism. ER stress triggers a cascade of signaling networks referred to as the unfolded protein response (UPR) to reduce stress and restore homeostasis. The UPR activates the cellular response that leads to the transcription of molecular chaperones to alleviate this stress response. Importantly, three major ER proteins, inositol-requiring enzyme 1α (IRE1α, also known as ERN1), the PKR-like ER kinases (PERK), and activating transcription factor 6α (ATF6α) have been shown to bind to misfolded or unfolded proteins to initiate the UPR. Activation of the UPR is necessary to reduce stress; however, insufficient clearance of these misfolded proteins or prolonged activation of the pathways that induce ER stress result in apoptotic cell death (Chen et al., 2015; Endo et al., 2006; Ron and Walter, 2007).

ER stress activates the inflammatory processes, especially in macrophages, which are the sentinels of the immune systems (Ho et al., 2012). These specialized cells, called first responders, play major roles in the maintenance of tissue integrity or tissue damaging signals, host defense and inflammation. Disturbances in this delicate balance cause the cells to be more susceptible to activation of inflammatory processes, leading to immune diseases and disorders (Franken et al., 2016; Gautier et al., 2012; Lavin et al., 2015). Macrophages are key components of the innate immune system and are crucial parts of a system that senses and responds to tissue invasion by infectious microorganisms and tissue injury through various scavenger, pattern recognition and phagocytic receptors (Lavin et al., 2015). Although the exact mechanism for the activation of macrophages under ER stress is not entirely understood, Ca^2+^ could be the vital link. In addition, switching of the macrophages to the pro-inflammatory M1 phenotype is the key to chronic immune activation.

The aim of this study was to identify the pathways that involve ER stress and lead to immune activation. Our data show that Ca^2+^ homeostasis is vital to macrophages activation. We report for the first time that ER stress inducers lead to the loss of Ca^2+^ entry, which corresponds to a decrease in ER Ca^2+^ levels, and precede the unfolded protein response. Loss of ER and intracellular Ca^2+^ was primarily due to the decrease in expression of Orai1, TRPC1 and STIM1. Orai1 is a pore-forming component of I_CRAC_, and STIM1 is an ER-Ca^2+^ sensor, which activates SOCE. TRPC1 and Orai1, together with STIM1, produce Ca^2+^ signals that consequently regulate cell functions. We further report that the endogenous Ca^2+^ entry channel in macrophages cells is dependent on a TRPC1–Orai1 complex, and that loss of TRPC1–Orai1, function induces abnormal UPR and the induction of ER stress. Furthermore, loss of Ca^2+^ entry increased the pro-inflammatory M1 phenotype, induced cytokine production, and increased phagocytosis. Taken together, these results suggest that loss of Ca^2+^ signaling initiates ER stress, and that restoration of Orai1–STIM1 complex function is essential for inhibiting ER stress and cytokine production, which regulates inflammation. These results not only help in understanding the physiology of ER stress-related disorders but could help in the development of novel therapeutic modalities for ER stress-related disorders by improving our comprehension of the relationship between ER stress and the UPR processes.

## RESULTS

### Treatment with tunicamycin or brefeldin A induces ER stress, affecting cell proliferation of macrophages

**Fig. 1.**
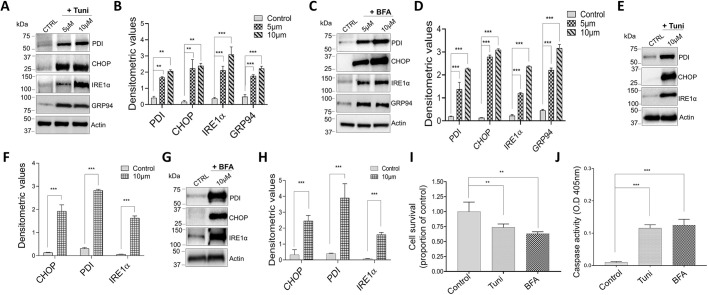
**Tunicamycin** **and brefeldin A treatment induces ER stress-causing cell death in macrophages.** (A,C) Representative immunoblot images showing the expression of ER stress markers PDI, CHOP, IREα and GRP94, and of actin, in Raw 264.7 macrophages pretreated with 5 μM or 10 μM tunicamycin (Tuni) (A), or BFA (C) for 12 h. (B,D) Quantification of densitometric values (mean±s.d.) from bands as shown in A and C. (E,G) Representative immunoblot images showing the expression of ER stress markers in bone marrow-derived macrophages (BMDMs) pretreated with 10 μM Tuni (E) or BFA (G) for 12 h. (F,H) Quantification of densitometric values (mean±s.d.) from bands as shown in E and G. (I,J) Quantification (mean±s.d.) of cell survival (I) and caspase activity (J) of Raw 264.7 cells pretreated with 10 μM BFA or Tuni for 12 h. **P<0.01, ***P<0.001 by one-way ANOVA test. The data shown are representative of three independent experiments.

ER stress response could trigger an inflammatory response, therefore causing metabolic issues in the cell (Cao and Kaufman, 2014; Grootjans et al., 2016). Thus, we initiated our study by assessing the response to well-known ER stress inducers tunicamycin (Tuni), a known glycosylation suppressor, and brefeldin A (BFA), an inhibitor of Golgi complex transportation in the macrophage cell line. Raw 264.7 cells were incubated at different concentrations of both Tuni and BFA (5 µM and 10 µM) for 6 and 12 h. After treatment, proteins were extracted, and western blots were performed, using β-actin as positive control. Importantly, cells treated with 5 µM Tuni for 12 h showed an increase in the expression of ER stress markers, whereas, treatment for 6 h did not show a similar increase in CHOP (also known as DDIT3) or other ER stress proteins (Fig. S1A). Expression of molecular chaperone GRP94 (also known as HSP90B1), was significantly increased, along with increased expression of PDI (also known as P4HB), IRE1α and CHOP proteins in the presence of Tuni at 12 h of treatment (Fig. 1A,B). Increasing Tuni concentration, however, showed a dose-dependent increase in the expression of ER stress markers, whereas no change in the actin level was observed (Fig. 1A,B; Fig. S1A). Consistent with these results, when exposed to BFA Raw 264.7 cells also showed an increase in ER stress markers, where an increase in the expression of CHOP, GRP94 and other ER stress proteins was observed in cells treated with a higher concentration of BFA for 12 h (Fig. 1C,D; Fig. S1B). 

To further confirm our cell line results, we isolated mouse primary macrophages to investigate whether ER stress markers are also increased upon the addition of Tuni and BFA. Our data again showed that when we treated primary bone marrow-derived macrophages (BMDMs) with either Tuni or BFA, expression of ER stress markers was increased within 12 h of treatment. (Fig. 1E–H). Importantly, treating for a longer time (16 h) significantly decreased the cell number of primary macrophage (data not shown), suggesting that both Tuni and BFA lead to a chronic unfolded protein response, resulting in cell death. Thus, we also evaluated the consequence of this ER stress and tested cell viability where cells were exposed to Tuni or BFA treatments. Interestingly, our data showed that 6 h of Tuni or BFA treatment did not induce cell death and no significant difference in cell viability was observed (data not shown). In contrast, a significant increase in cell death was observed after prolonged treatment (12 h) with 10 µM of either Tuni or BFA (Fig. 1I). Additionally, incubating for 24 h with either Tuni or BFA further increased cell death and the majority of cells were dead within 24 h of treatment (data are shown). To identify how ER stress induces cell death, caspase 3 activity was measured, and was shown to be significantly increased in cells that were treated with 10 µM (12 h) of either Tuni or BFA (Fig. 1J). Analyzed together, these results show that when ER stress is induced in macrophages, it causes upregulation of known markers of the UPR, which leads to chronic activation of caspase 3, and thereby triggers apoptosis.

### Treatment with tunicamycin or brefeldin A decreases ER and cytosolic Ca^2+^

**Fig. 2.**
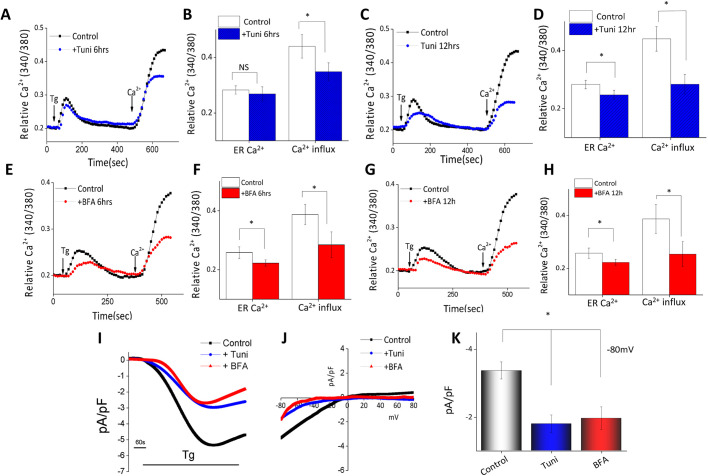
**Tunicamycin and brefeldin A treatment decreased the Ca^2+^ currents in murine macrophages.** (A,C,E,G) Ca^2+^ tracing was performed in Raw 264.7 cells following treatment with 10 μM Tuni for 6 h (A) or 12 h (C), or 10 μM BFA for 6 h (E) or 12 h (G). Representative traces of the analog plots of the fluorescence ratio (340/380) from an average of 40–60 cells are shown. (B,D,F,H) Quantification (mean±s.d.) of fluorescence ratio (340/380) under conditions as shown in A,C,E,G, as labeled in the figure. (I) Whole-cell patch recording showed that bath application of 1 µM Tg induced an inward-rectifying current in Raw 264.7 cells. (J,K) Average IV curves (J) and current density (K) from 6–9 cells at −80 mV under conditions as shown in I. *P<0.05 by one-way ANOVA test; NS, non-significant.

To establish the mechanism by which ER stress is initiated, we evaluated Ca^2+^ signaling in BMDMs. Addition of thapsigargin (Tg) (1 µM in Ca^2+^-free buffer), a SERCA pump blocker that depletes ER Ca^2+^, caused a small increase in intracellular Ca^2+^ ([Ca^2+^]_i_) levels (first peak) in BMDMs (Fig. 2A–H). In the presence of 1 mM external Ca^2+^, cells showed a significant increase in [Ca^2+^]_i_ levels (second peak), indicating the presence of store-mediated Ca^2+^ entry (Fig. 2A–H). Importantly, cells treated with Tuni (10 µM for 6 h) to induce ER stress were shown to have a significant reduction in SOCE without any change in internal ER Ca^2+^ release (Fig. 2A,B). In contrast, when Tuni treatment (10 µM) continued for 12 h, not only was Ca^2+^ entry significantly decreased, but also the internal ER Ca^2+^ level (Fig. 2C,D). Consistent with these results, addition of BFA (10 µM for 6 h), also revealed a significant decrease in Ca^2+^ entry; whereas 12 h treatment led to a decrease in both ER Ca^2+^ level and Ca^2+^ entry, respectively (Fig. 2E–H). To establish the molecular identity of the Ca^2+^ influx channel in macrophages, electrophysiological recordings of membrane currents were performed. The addition of Tg induced an inward current, which was partially inward-rectifying in nature and reversed between 0 and −5 mV (Fig. 2I,J). The current properties observed were mixed as previously observed with TRPC1 and Orai1 channels (Liu et al., 2004; Selvaraj et al., 2012; Shi et al., 2012; Yuan et al., 2007). Moreover, in the presence of Tuni or BFA, Tg-induced Ca^2+^ currents were significantly inhibited in Raw 264.7 cells (Fig. 2I–K). Taken together, these results suggest that in macrophages, the addition of ER stress inducers (Tuni, BFA) leads to a loss of SOCE that further decreases ER Ca^2+^ levels, thereby leading to ER stress. Importantly, Ca^2+^ entry was dependent on TRPC1 and Orai1 channels, and Tuni- and BFA-induced loss of Ca^2+^ entry precedes the expression of ER stress markers. 

### Ca^2+^ channels are downregulated by treatment with tunicamycin or brefeldin A

**Fig. 3.**
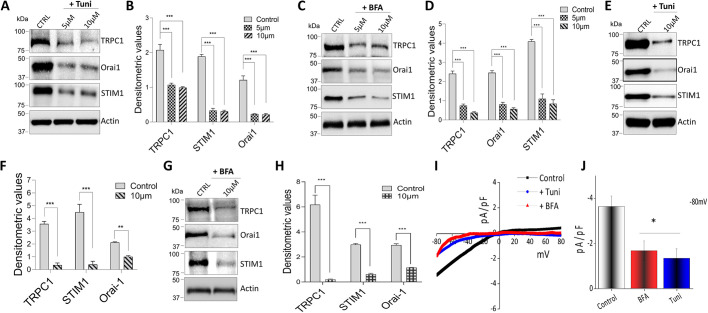
**Tunicamycin and brefeldin A treatment decrease Ca^2+^ channel expression in murine macrophages.** (A,C) Representative immunoblots showing the decreased expression of Ca^2+^ entry channels proteins TRPC1, Orai1 and STIM1 in Raw 264.7 macrophages pretreated with 5 μM or 10 μM Tuni (A) or BFA (C) for 12 h. (B,D) Quantification of densitometeric values (mean±s.d.) from bands as shown in A and C. (E,G) Representative immunoblot images showing the decreased expression of Ca^2+^ entry channels in BMDMs pretreated with 10 μM Tuni (E) or BFA (G) for 12 h. (F,H) Quantification of densitometric values (mean±s.d.) from bands as shown in E and G. (I,J) Whole-cell patch recording showing average IV curves (I) and current intensity (J) from 6–9 cells at −80 mV of BMDMs pretreated with 10 μM BFA or Tuni for 12 h. *P<0.05, **P<0.01, ***P<0.001 by one-way ANOVA test; NS, non-significant. The data shown are representative of three independent experiments.

To further understand how Ca^2+^ influx is inhibited in macrophages, we decided to investigate the expression of the Ca^2+^ channel proteins themselves. Raw 264.7 cells were again exposed to Tuni or BFA for 6 or 12 h and the expression of SOCE channel proteins was observed. Importantly, cells pretreated with either 5 or 10 µM of Tuni resulted in a significant downregulation of TRPC1 and Orai1 (Fig. 3A,B). In addition, activity of STIM1, which modulates both TRPC1 and Orai1, was also significantly decreased, with no observed changes in actin levels (Fig. 3A,B). Similar results were also observed with BFA, with significant decreases in TRPC1, Orai1 and STIM1 expression levels (Fig. 3C,D). Importantly, cells treated with lower doses of either Tuni or BFA for 6 h did not show a significant decrease in TRPC1, STIM1 or Orai1 protein levels (Fig. S1C,D). Taken together, these results suggest that STIM1 and Orai1 are important Ca^2+^ entry channels in macrophages, along with the TRPC1 channel, as they are all decreased during the induction of ER stress. These results are consistent with a previous study wherein membrane depolarization was not enough to generate cytosolic Ca^2+^ signals in immune cells (Demaurex and Nunes, 2016), suggesting that Ca^2+^ signaling is mostly via the SOCE mechanism. To further confirm these findings, we studied the expression of SOCE components on primary BMDMs (Fig. 3E–H) treated with Tuni or BFA (10 µM each) for 12 h. Confirming our cell line results, the expression of TRPC1, Orai1 and STIM1 was also decreased. Additionally, electrophysiology was performed on BMDMs (Fig. 3I,J), and in further support of our cell line data, a decrease in Ca^2+^ currents was observed when primary cells were treated with Tuni or BFA. The findings of this study so far have established a direct relationship between Ca^2+^ levels and ER stress on the regulation of cell survival in macrophages. 

### SKF96365 treatment induces ER stress in macrophages cells

**Fig. 4.**
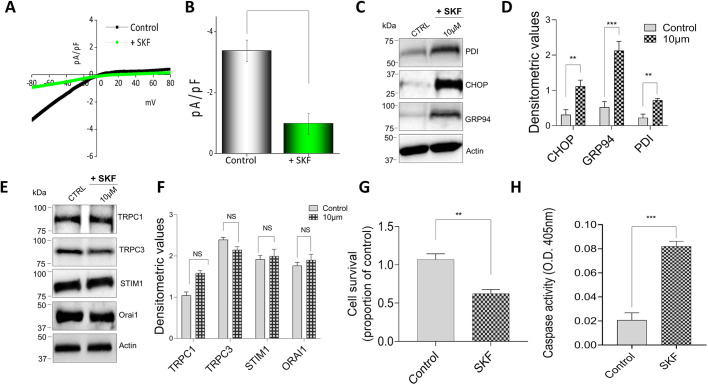
**ER stress is induced by SKF96365 pretreatment on the murine macrophage cell line.** (A,B) Whole-cell patch recording showing average IV curves (A) and current intensity (B) from 6–9 cells at −80 mV in Raw 264.7 macrophages pretreated with 10 μM SKF96365 (SKF) for 12 h. (C) Representative immunoblot images showing the induction of ER stress as seen in the increased expression of marker proteins in Raw 264.7 macrophages pretreated with 10 μM SKF for 12 h. (D) Quantification of densitometric values (mean±s.d.) from bands as shown in C. Immunoblots representing the expression of Ca^2+^ entry channel proteins TRPC1, TRPC3, Orai1 and STIM1 in Raw 264.7 macrophages pretreated with 10 μM SKF for 12 h. (F) Quantification of densitometric values (mean±s.d.) from bands as shown in E. (G,H) Quantification (mean±s.d.) of cell survival (G) and caspase activity (H) of Raw 264.7 cells pretreated with 10 μM SKF for 12 h. **P<0.01, ***P<0.001 by one-way ANOVA test; NS, non-significant. The data shown are representative of three independent experiments.

We next evaluated the physiological effect of blocking SOCE channels on ER stress. SKF96345 (SKF), a well-established Ca^2+^ entry blocker that blocks both TRPC1 and Orai1 channels (Sukumaran et al., 2018), was used. Importantly, exposing Raw 264.7 cells to SKF (10 µM for 12 h) resulted in a significant decrease in Tg-induced Ca^2+^ currents (Fig. 4A,B). Consistent with the loss of SOCE, a significant increase in levels of ER stress markers such as CHOP, GRP94 and PDI, but not actin, was observed in cells that were pretreated with SKF (10 µM for 12 h) (Fig. 4C,D). We next evaluated whether SKF treatment also decreased the expression of SOCE channel proteins as observed in Fig. 3. Importantly, no change in TRPC1, STIM1 or Orai1 expression was observed upon SKF treatment (Fig. 4E,F), suggesting that the loss of Ca^2+^ entry was the reason for the induction of the ER stress response. Furthermore, we observed that when macrophages were treated with SKF, it also resulted in a significant decrease in cell survival (Fig. 4G), along with an increase in caspase 3 activity (Fig. 4H). These data showed that an increase in ER stress is directly related to inhibiting SOCE channel activity, which leads to a decrease in ER Ca^2+^ levels and induces cell death in macrophages. 

### Treatment with tunicamycin, brefeldin A or SKF96365 induces cell autophagy and apoptosis

**Fig. 5.**
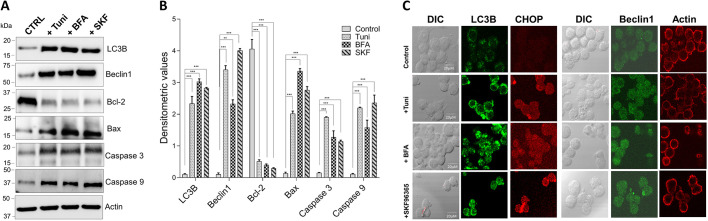
**Tunicamycin, brefeldin A and SKF96365 treatment induce autophagy and apoptosis in Raw 264.7 macrophages.** (A) Representative immunoblot images showing expression of markers for the induction of autophagy and apoptosis in Raw 264.7 macrophages treated with 10 µM Tuni, BFA or SKF for 12 h. (B) Quantification of densitometric values (mean±s.d.) from bands as shown in A. (C) Representative microscopy images of Raw 264.7 macrophages stained for LC3B (green), CHOP (red) and DAPI (blue) after cells were exposed to 10 µM Tuni, BFA or SKF for 12 h. **P<0.01; ***P<0.001 by one-way ANOVA test. The data shown are representative of three independent experiments. Scale bars: 20 µm.

It has been established that cell death starts once ER stress is highly elevated in the cell. ER stress and autophagy appear to be initially involved in actions to protect the cell, but induce cell death under prolonged stress conditions (Rashid et al., 2015). Our data show that treatment with Tuni, BFA or SKF induces caspase activity, which leads to cell death. To establish the correlation between the increase in ER stress and autophagy, we decided to analyze the expression of known autophagy and apoptosis markers LC3B (active component), beclin1, Bcl-2, caspase 3, caspase 9 and Bax in Raw 264.7 cells. Importantly, an increase in the expression of autophagy markers, and of caspase 3, caspase 9 and Bax, was observed (Fig. 5A,B). Confocal images of cells treated with the ER stress inducers Tuni or BFA, or with Ca^2+^ channel inhibitor SKF, again showed an increase in autophagic vesicles (as observed with staining for LC3B or beclin1) and an increase in ER stress protein CHOP (Fig. 5C). Taken together, these data suggest that loss of Ca^2+^ channels, which is directly responsible for an increase in ER stress, causes a chain reaction that triggers an increase in autophagy, resulting in apoptosis of macrophages. 

### Treatment with tunicamycin, brefeldin A or SKF96365 enhances the secretion of cytokines and causes cell cycle arrest

**Fig. 6.**
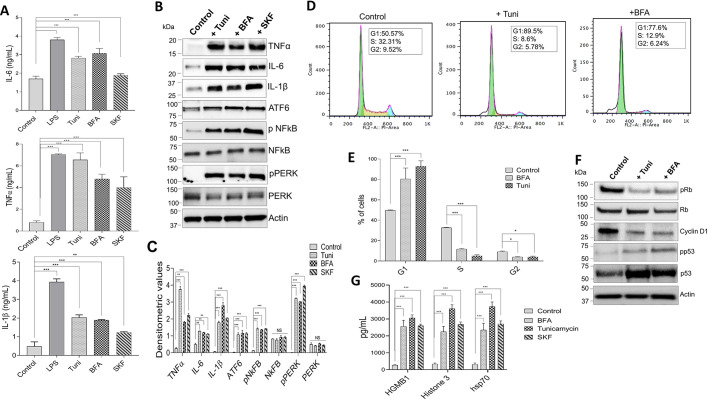
**ER stress induces macrophages to produce mature pro-inflammatory cytokines.** (A) Raw 264.7 macrophages were treated with DMSO or 10 µM of ER stress inducers Tuni, BFA or SKF for 12 h. Levels of pro-inflammatory cytokines IL-6, TNFα and IL-1β in cell supernatants were measured using ELISA. LPS was used as a positive control. (B) Representative immunoblots showing expression levels of UPR pathway proteins and NF-κB in cell extracts from Raw 264.7 macrophages treated with ER stress-inducing drugs or DMSO. (C) Quantification of densitometric values (mean±s.d.) from bands as shown in B. (D) Typical DNA content frequency histograms representing Raw 264.7 cells treated with DMSO, Tuni or BFA. The cells were stained with PI and fluorescence was measured. (E) Percentage of cells in each cell phase for the treatment groups as shown in D. (F) Representative immunoblots showing expression levels of cell cycle proteins in cell lysates from Raw 264.7 macrophages treated with 10 µM Tuni or BFA for 12 h. (G) ELISA results showing increased expression of HMGB1, Hsp70, and histone 3 when Raw 264.7 macrophages were treated with DMSO, Tuni, BFA or SKF. Data are representative of three independent experiments. *P<0.05, **P<0.01, ***P<0.001 by one-way ANOVA test; NS, non-significant. The data shown are representative of three independent experiments.

Following our findings, we focused on the effects of the treatments on the production of inflammatory cytokines under conditions of ER stress. It has been established that cytokines play critical roles in host defense against pathogens; however, when produced in high demand, they may also be responsible for pathological inflammation (Brozzi et al., 2015; Smith, 2018; Vaeth et al., 2015). Both Raw 264.7 and BMDM cells were treated with 10 µM of Tuni, BFA or SKF96365 for 12 h and cytokine levels were analyzed. We observed that release of the major cytokines interleukin-6 (IL-6), interleukin 1 beta (IL-1β) and tumor necrosis factor alpha (TNF-α) were all significantly increased (Fig. 6A; Fig. S2A). Similarly, intracellular levels of these cytokines were also increased in the presence of all three treatment agents. Moreover, expression of proteins involved in three UPR pathways, IRE1α (data not shown), PERK and ATF-6 (Hotamisligil, 2010), along with the activation of NF-κB, were increased in the presence of these stressors (Fig. 6B,C). It has previously been established that all three UPR pathways impact the activation of NF-κB, which is transported to the nucleus in response to immune signaling, where it triggers inflammatory cytokines such as IL-6 and TNF-α. (Hayden and Ghosh, 2008; Smith, 2018). 

Cell cycle progression was evaluated next, where Raw 264.7 cells were treated with various ER stress inducers, and cell cycle distribution was analyzed by measuring the DNA content using a flow cytometer. The results of the cell cycle analysis clearly showed that addition of Tuni or BFA inhibited cell cycle progression, which is consistent with our cell viability data. Importantly, Tuni and BFA treatments induced a significant increase in the number of cells in the G1 phase, with a subsequent decrease in the number of cells in the S and G2 phase (Fig. 6D,E), indicating inhibition of cell cycle progression. To further corroborate our cell cycle findings, western blotting demonstrated that expression of proteins associated with the cell cycle, such as cyclin D1, phosphorylated Rb (Rb is also known as RB1) and phosphorylated p53 (also known as TP53), was decreased (Fig. 6F). Studies have shown that cyclin D1, along with Rb, are limiting for progression of the cell cycle, and hence their inhibition results in G1 cell cycle arrest (Brewer and Diehl, 2000). These data agree with studies which showed that the UPR is responsible for the coordination of the induction of ER chaperones, while at the same time decreasing the proteins synthesis, promoting growth arrest in the G1 phase of the cell cycle (Brewer and Diehl, 2000; Han et al., 2013). Damage-associated molecular patterns (DAMPs) are known to play a role in inflammatory response and influence adaptive immunity by activating several types of innate immune machinery, including inflammasomes (Land, 2015). Furthermore, it has been shown previously that HSP70 is responsible for the activation of monocytes (Pockley et al., 2008). To evaluate the expression of DAMPs, we chose to investigate the following representatives of the group: high-mobility group box 1 (HMGB1), heat-shock protein 70 (HSP70) and histone 3. Importantly, increased expression of all the DAMPs was observed when we treated Raw 264.7 cells with Tuni, BFA or SKF (Fig. 6G), suggesting that loss of Ca^2+^ entry leads to the secretion of DAMPs that increase immune function.

### ER stress inducers promote the M1 phenotype and stimulate phagocytic activity

**Fig. 7.**
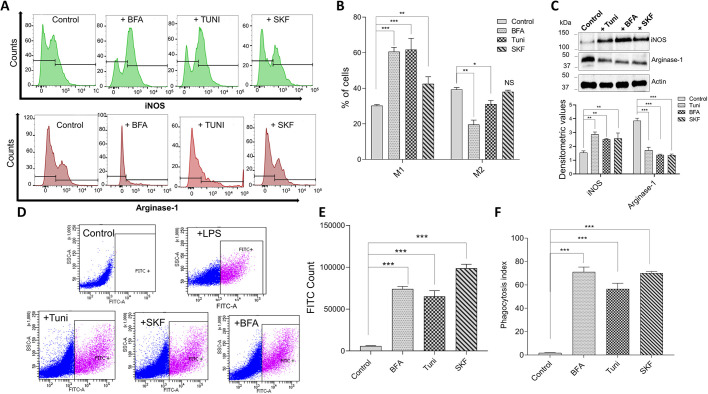
**ER stress induces macrophage polarization and controls macrophage phagocytic action.** (A) Validation of M1 and M2 polarization using flow cytometry. Raw 264.7 macrophages were treated with 10 μM Tuni, BFA or SKF for 12 h and stained for iNOS and arginase-1, which are specific surface markers of M1 and M2 macrophages, respectively. (B) Percentages of iNOS- and arginase-1-positive cells for each treatment as shown in A. (C) Representative immunoblots showing expression levels of iNOS and arginase-1 in cell extracts from Raw 264.7 macrophages treated with 10 μM Tuni, BFA or SKF for 12 h. Quantification of densitometric values (mean±s.d.) from bands are shown below. (D) Flow cytometric evaluation of latex beads (IgG–FITC complex) in Raw 264.7 cells treated with Tuni, BFA or SKF (10 μM for 12 h) and analyzed for FITC fluorescence. LPS was used as a positive control. (E) Quantification of numbers (mean±s.d.) of FITC-positive cells as shown in D. (F) Raw 264.7 macrophages were incubated with FITC-conjugated latex beads for 2 h hours following treatment with Tuni, BFA or SKF (10 μM for 12 h), and phagocytic activity was measured using fluorescent microscopy. The mean±s.d. phagocytic index was calculated for each treatment group (percentage of cells with engulfed particles multiplied by the mean number of particles engulfed per cell). *P<0.05, **P<0.01, ***P<0.001 by Student's *t*-test; NS, non-significant. The data shown are representative of three independent experiments.

Studies have shown that the phagocytosis of pathogens by macrophages is a crucial function of innate immune responses (Santoni et al., 2018; Hirayama et al., 2017). To further understand the effects of ER stress inducers on the activation of macrophages and consequently on their polarization, cells were again treated with ER stress inducers and their macrophage function was evaluated. Importantly, our data showed that ER stress activates macrophages, and leads to an increase in numbers of M1 type macrophages. Stimulation of Raw 264.7 cells with IFNγ for 24 h in the presence of Tuni, BFA or SKF revealed a significant increase in the expression of known M1 marker iNOS (also known as NOS2) (Fig. 7A,B). Consistent with this, a subsequent decrease in the M2 phenotype (evaluated using marker arginase-1) was observed (Fig. 7A,B). For further confirmation of macrophage polarization, we evaluated the expression of iNOS and arginase-1 when Raw 264.7 cells were treated with Tuni, BFA or SKF without stimulation with IFNγ, which again led to an increase in iNOS expression and a decrease in arginase-1 when compared to the cells treated with DMSO (Fig. 7C). We also used other classic M1/2 markers, such as CD80_low_/CD206_high_, which again showed that the M2 phenotype was decreased in the presence of ER stress inducers (Fig. S2B). 

To verify how ER stress influences macrophage function, phagocytic activity in the Raw 264.7 macrophage cell line was evaluated. Phagocytosis assays were performed using flow cytometry or microscopy with FITC-labeled latex beads, which revealed a significant increase in levels of phagocytosis, as well as total FITC count, in cells pretreated with Tuni, BFA or SKF (Fig. 7D–F). Importantly, treatment with LPS, which was used as a positive control, also led to an increase in phagocytosis. In conclusion, our data suggest that ER stress promotes the M1 phenotype, which induces increased cytokine levels and activation of phagocytosis.

### Overexpression of Orai1 and STIM1 allows recovery of the macrophage cell line by decreasing ER stress protein levels

**Fig. 8.**
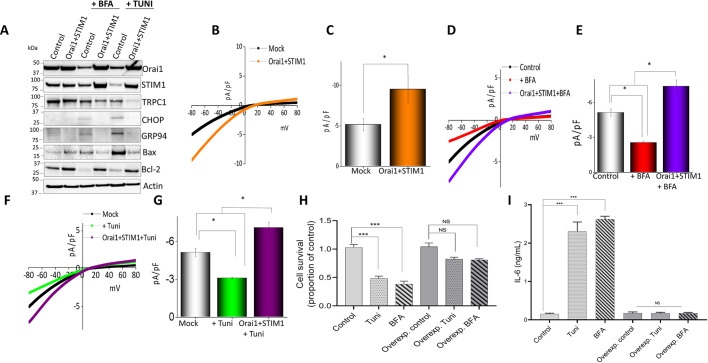
**Overexpression of calcium channel proteins Orai1 and STIM1 rescues macrophages from ER stress and cell death.** (A) Representative immunoblots showing expression of ER stress and apoptosis markers in control (mock) Raw 264.7 cells or cells overexpressing Orai1 and STIM1, pretreated with Tuni or BFA (10 μM for 12 h). (B–G) Whole-cell patch recording showing average IV curves (B,D,F) and current intensity (C,E,G) of 6–9 cells at −80 mV under conditions as indicated in Raw 264.7 macrophages. (H) Quantification (mean±s.d.) of cell survival in control Raw 264.7 cells or cells overexpressing Orai1 and STIM1, pretreated with 10 µM Tuni or BFA for 12 h. (I) ELISA results showing mean±s.d. IL-6 levels in supernatants of Raw 264.7 macrophages treated with DMSO, Tuni or BFA. *P<0.05, ***P<0.001 by one-way ANOVA test; NS, non-significant. The data shown are representative of three independent experiments.

After observing the importance of SOCE in modulating ER stress, we next evaluated whether overexpression of Orai1 and/or STIM1 would inhibit ER stress and induce cell survival. Raw 264.7 cells overexpressing Orai1 and STIM1 showed a decrease in ER stress markers when treated with Tuni or BFA (Fig. 8A). Densitometric values for individual blots showing the expression of the proteins are given in Fig. S3. Accordingly, overexpression of Orai1 and STIM1 restored levels of Ca^2+^ entry that had been decreased by Tuni or BFA treatment (Fig. 8B–G). Cell survival assays also revealed a significant decrease in Tuni- or BFA-induced cell death in cells overexpressing Orai1 and STMI1 (Fig. 8H). Importantly, a significant decrease in pro-inflammatory cytokines IL-6, IL-1β and TNFα (Fig. 8I; Fig. S4A) was observed in both Raw 264.7 and primary macrophages, indicating that restoration of Ca^2+^ entry could prevent ER stress-induced cytokine release. To further establish whether loss of TRPC1, Orai1 or STIM1 expression would also induce pro-inflammatory cytokine release, we also investigated the release of pro-inflammatory cytokines in cells where the expression of TRPC1, Orai1 or STIM1 was inhibited. Importantly, silencing of TRPC1, Orai1 or STIM1 further increased cytokine levels (Fig. S4B). Taken together, these results clearly show that Orai1- and/or STMI1-mediated SOCE is critical for Ca^2+^ signaling in non-excitable cells and is essential for ER stress-induced abnormal activation of immune cells and cell survival. 

## DISCUSSION

Incidence of chronic inflammation is associated with several diseases such as rheumatoid arthritis, systemic lupus erythematosus and multiple sclerosis (Hunter, 2012). Consequently, scientific inquiry into the mechanisms underlying prolonged immune activation has gained interest. Although an array of diseases are linked to alterations in Ca^2+^ homeostasis, the study of Ca^2+^ signaling in modulating ER stress has been underrepresented. Evidence suggests that in macrophages, [Ca^2+^]_i_ is mediated by the store-operated Ca^2+^ entry (SOCE) mechanism (Chauhan et al., 2018; Tian et al., 2016). SOCE was first identified as a major component of non-excitable cells (Parekh and Putney, 2005), but further research has identified SOCE within a multitude of tissue types (Woo et al., 2018; Prakriya and Lewis, 2015). It has further been determined that STIM1 and Orai1 proteins are critical mediators of SOCE and might also modulate immune cell functions (Shaw and Feske, 2012b; Vaeth et al., 2015). Thus, we focused on identification of the endogenous Ca^2+^ entry channel and establishing its role in macrophage function, specifically in ER stress conditions.

In our study, we identify that TRPC1 and Orai1 are major regulators of ER-stress induced immune activation. We initially showed that in macrophages, when Ca^2+^ entry is inhibited by the action of stress-inducing agents, it directly leads to the activation of ER stress and promotes cell death. Importantly, increased expression of CHOP, which is a multifunctional transcription factor in the ER stress response and plays a major role in the promotion of cell death, and increased activation of IRE1α, a known protein involved in the regulation of the UPR (Li et al., 2014; Sano and Reed, 2013), were observed. Interestingly, addition of either Tuni or BFA, which initiate ER stress, albeit through different mechanisms, decreased SOCE protein components, suggesting that this might be the common pathway. Activation of the UPR releases a cascade of events that promote the phosphorylation of NFκB and consequently move it to the nucleus. This in turn activates the expression of pro-inflammatory cytokines such as TNFα, IL-6 and IL-1β, which activate downstream mediators of inflammation (Hotamisligil, 2010). We showed that when the UPR is activated, it promotes ER stress and increases cell death. Our results provide evidence that the loss of Ca^2+^ influx induces ER stress levels in macrophages. Moreover, we showed that SOCE channels are essential for Ca^2+^ entry and for cell survival, which can be relevant in autoimmune diseases. Alongside TRPC1, Orai1 and STMI1 were shown to have an important role in Ca^2+^ homeostasis. Basic macrophage functions such as phagocytosis and macrophage polarization were also affected by the activation of ER stress.

Macrophages are responsible for detecting, engulfing and destroying pathogens and apoptotic cells. They are also the first line of defense that phagocytose pathogens and initiate the cellular immune response (Freeman and Grinstein, 2014; Martin et al., 2014). Interestingly, Ca^2+^ is critical for the engulfment of phagocytes, and impaired Ca^2+^ influx could be responsible for the failure of the anti-inflammatory response, which could lead to inflammation and autoimmunity (Gronski et al., 2009). Any change in Ca^2+^ influx could result in abnormal function and affect the regulation of immune functions, such as the activation of pro-inflammatory cytokines TNF-α, IL-6 and IL-1β, which are involved in the upregulation of inflammatory reactions. Consequently, decreases in expression of the Ca^2+^ channel proteins could be associated with an abnormal immune response.

Our data show that loss of function of TRPC1 and/or Orai1 causes a decrease in Ca^2+^ influx, leading to autophagy and cell death. These findings are consistent with previous studies showing that intracellular Ca^2+^ is widely recognized as a key regulator of autophagy (Parys et al., 2012) and apoptosis (Sano and Reed, 2013; Yoshino et al., 2017). Therefore, a decrease in the expression of Ca^2+^ channels in macrophages can be strongly associated with cell death, as demonstrated by our results. Moreover, Ca^2+^ signaling regulates cell cycle progression in various cell types. Ca^2+^ transients are detected at wakening from quiescence, at the G1/S shift, during the S-phase and after mitosis (Chen et al., 2016). The activation of SOCE channels indeed regulates cell-cycle protein levels and controls the G1/S cell cycle transition. Loss of Ca^2+^ influx prevents the progression of cells into the cell cycle, causing cell cycle arrest that could further lead to apoptosis. Consistent with those studies, we demonstrated that macrophages under ER stress show a loss of SOCE, which further decreases the expression of cell cycle proteins associated with cell cycle arrest. Consistent with these results, overexpression of Orai1 and/or STIM1 seems to restore Ca^2+^ influx in these cells. We also observed an increase in cell survival, as it is characterized by a decrease in ER stress protein expression, and observed a reverse in the induction of apoptosis and the production of pro-inflammatory cytokines. Taken together, our results could indicate that expression of SOCE channels Orai1 and/or TRPC1 plays a major role in modulating ER stress and, consequently, inducing inflammation by immune activation via the UPR pathway.

## MATERIALS AND METHODS

### Cell culture, primary cells and reagents

The murine Raw 264.7 cell line was obtained from Sigma-Aldrich, tested for mycoplasma contamination and maintained at 37°C with 5% CO_2_ in Dulbecco's modified Eagle's medium (DMEM) supplemented with 10% fetal bovine serum (Gibco) with streptomycin and penicillin. As the cells were from a commercial source we did not authenticate them prior to our study. Tunicamycin (Tuni), brefeldin A (BFA), thapsigargin and SKF96365 (SKF) were purchased from Sigma-Aldrich and used at the indicated concentrations. For the primary cells, bone marrow-derived macrophages (BMDMs) were isolated from the femurs of C57BL/6 mice (6 weeks old, male) (Jackson's laboratory and bred in our animal facility). The animal protocol was approved by the University of Texas Health San Antonio IACUC committee. Femurs were flushed out with PBS using a sterile needle. The BMDM cells were cultivated in DMEM medium (Life Technologies) containing L929 conditioned media. After 5 days, fully differentiated BMDMs had formed on the bottom of the culture plates. The isolated macrophages were then incubated at 37°C in DMEM medium supplemented with 10% FBS and 1% penicillin/streptomycin solution (Trouplin et al., 2013; Weischenfeldt and Porse, 2008).

### Overexpression of Orai1 and STIM1

Orai1 and STIM1 adenovirus were purchased from AbmGood (Richmond, BC, Canada), and were amplified in HEK 293 cells (ATCC) using the manufacturer's protocol to obtain the crude viral lysate. For overexpression experiments, 100 μl of the crude viral lysate encoding Orai1 or STIM1 was added to 75% confluent cells in a 6-well dish. Gene transduction was evaluated 24 h after transduction using western blotting. For silencing experiments respective siRNA (siOrai1, assay ID: 288184; iTRPC1, assay ID: 187432; both Thermo Fisher Scientific) was used, and the transfection was performed with Lipofectamine RNAiMAX Reagent (Thermo Fisher Scientific) following the manufacturer’s protocol. Cells were seeded to be 70% confluent, and the Lipofectamine reagent was diluted to an optimal concentration (25 pmol per well in a 6-well dish) in Opti-MEM medium (Gibco). We added the Lipofectamine to the diluted siRNA in a 1:1 ratio and incubated for 5 min at room temperature, after the stipulated time the complex was layered on the cells for 5 h, followed by adding fresh media. Transfected cells were then used for individual experiments.

### Cell viability assay

Cell viability was measured using 3-[4,5-dimethylthiazol-2-yl]-2,5-diphenyltetrasolium bromide (MTT) (Sigma-Aldrich) according to the manufacturer's protocol. Primary BMDMs were seeded in 96-well plates at a density of 0.5×10^5^ cells/well in DMEM supplemented with 10% FBS, and streptomycin and penicillin. The cultures were incubated for 24 h at 37°C with 5% CO_2_, followed by the addition of fresh medium prior to the experiment being performed. Cell viability was measured using the MTT method. 10 μl of MTT reagent (5 mg/ml MTT in PBS) was added to each well and incubated in a CO_2_ incubator for 4 h. The resulting formazan dye was extracted with 100 μl of 0.01 N HCl in isopropanol, and within an hour, the absorbance was measured on an ELISA plate reader with a test wavelength of 570 nm and a reference wavelength of 630 nm. Cell viability of experimental cultures was expressed as a percentage of the control culture.

### Calcium measurement and electrophysiology

For fluorescence measurements, cells were incubated with 2 μM Fura-2 (Molecular Probes) for 45 min and then washed twice with Ca^2+^-free SES buffer [in mM, NaCl, 145; KCl, 5; MgCl_2_, 1; HEPES, 10; glucose, 10; pH 7.4 (NaOH); reagents from Sigma-Aldrich]. Cells were monitored with a charge-coupled device (CCD) camera-based imaging system. Images of multiple cells collected at each excitation wavelength were processed using C*IMAGING Systems - SimplePCI (Compix Inc.) to provide ratios of Fura-2 fluorescence from excitation at 340 nm to that from excitation at 380 nm (F340/F380) as previously described (Sun et al., 2014). For patch-clamp experiments, coverslips with cells were transferred to the recording chamber and perfused with an external Ringer's solution with the following composition: 145 mM NaCl, 5 mM KCl, 1 mM MgCl_2_, 1 mM CaCl_2_, 10 mM HEPES, 10 mM glucose, pH 7.4 (NaOH). The patch pipette had resistances between 3–5 m after filling with the standard intracellular solution that contained the following: 145 mM cesium methanesulfonate, 8 mM NaCl, 10 mM MgCl_2_, 10 mM HEPES, 10 mM EGTA, pH 7.2 (CsOH). The maximum peak currents were calculated at a holding potential of −80 mV. The I/V curves were made using a ramp protocol where current density was evaluated at various membrane potentials and plotted.

### Caspase 3 activity

Caspase 3 activity was measured using the Abcam Caspase 3 assay kit. 1×10^6^ cells were isolated using the cell lysis buffer provided and the liquid fraction was used to analyse the caspase 3 activity as per the manufacturer's instructions. The protocol is based on the formation of the chromophore p-nitroaniline (p-NA) by cleavage from the labeled substrate DEVD-pNA. Levels of p-NA can be quantified using a spectrophotometer reading absorbance at 400 or 405 nm.

### Protein extractions and western blotting

Whole-cell protein lysates were prepared in lysis buffer (10 mM Tris, 140 mM NaCl, 1% NP-40, 0.5% SDS, and protease inhibitors, pH 8.0), protein concentrations were determined using the Bradford reagent assay (Bio-Rad Laboratories), and 25–50 µg of proteins were resolved on NuPAGE Novex 4–12% BisTris gels, transferred to PVDF membranes. The membrane was blocked with skim milk in PBST solution and incubated with primary antibodies as listed below. After washing with PBST, secondary antibodies were applied and detected using the Clarity Western ECL Substrate and Clarity Max Western ECL Substrate (Bio-Rad Laboratories). Analysis and results were corrected for protein loading by normalization for β-actin expression. Densitometric analysis was performed using ImageJ software and results were corrected for protein loading by normalization for β-actin expression as previously described (Singh et al., 2004). Blot stripping was performed whenever necessary, using Restore PLUS Western Blot Stripping Buffer (Thermo Fisher). The blots were washed with PBS Tween 20, immersed in stripping buffer and incubated for 5–15 min at room temperature, then the stripping buffer was removed, the blot washed again and then blocked for another 30 min before incubation with a new antibody. The following primary antibodies were used for western blot analysis (all at 1:1000): anti-Orai1 (Thermo Fisher, PA5-26378), anti-TRPC1 (Alamone, ACC-010), anti-STIM1 (D88E10), anti-CHOP (L63F7), anti-PDI (C81H6), anti-GRP94 (D6X2Q), anti-NFκB p65 (p65) (8242S), anti-pNFκB p65 (pp65) (3033S), anti-Bax (D2E11), anti-IREI1α (14C10), anti-Bcl2 (D17C4), anti-ATF6 (D4Z8V), anti-PERK (C33E10), anti-pPERK (16F8), anti-TNFα (D2D4), anti-IL-6 (D5W4V), anti-IL-1β (D6D6T), anti-HMGB1 (D3E5), anti-Histone 3 (D1H2), anti-HSP70 (4872S), anti-Rb (D20), anti-pRB (D20B12), anti-Cyclin D1 (92G2), anti-p53 (1C12), anti-pp53 (Ser15), anti-LC3B (D11), anti-caspase 3 (9662S), anti-caspase 9 (C9) and anti-β-actin (4970S) (all from Cell Signaling Technology). HRP-conjugated goat anti-rabbit IgG (7074S) and goat anti-mouse IgG (7076S) (both from Cell Signaling Technology) were used as secondary antibodies.

### Measurement of cytokines levels

IL-6, TNF-α and IL-1β levels in the supernatant were measured using the appropriate enzyme-linked immunosorbent assay (ELISA) kit (Life Technologies) following the manufacturer's instructions, using standard diluent buffers designed for use with mouse serum. We used lipopolysaccharides (LPS, 100 ng/ml, Sigma-Aldrich) as a positive control, cells were tested following incubation for 3 h after treatments. HMGB1, histone 3 and HSP70 levels were measured by direct ELISA using a self-coating protocol, as previously described (Zhang et al., 2012). All samples were measured on a single 96-well plate for each cytokine, the cultured medium was centrifuged at 800 ***g*** for 5 min at 4°C and the supernatant was used as the sample. Based on that criterion, all cytokine values for the murine cell line Raw 264.7 and bone marrow-derived macrophages serum samples examined were above the limit of detection and within the reportable range of each assay.

### Confocal microscopy

For immunofluorescence staining, the cells were first fixed, then stained for the detection of specific antibodies, blocked with 10% donkey serum in phosphate-buffered saline, and probed for 1–2 h with anti-CHOP (Cell Signaling, L63F7), anti-LC3B (Cell Signaling, D11) and anti-Beclin1(Cell Signaling, D40C5) antibodies, all at 1:1000 at room temperature. Following incubation with primary antibodies, the cells were washed and reacted with fluorophore-conjugated respective secondary antibodies for 1 h at room temperature. Thereafter, the slides were washed again, and coverslip mounted using VECTASHIELD HardSet Mounting Media with DAPI (Vector Laboratories, Inc.). Images were acquired at 63× magnifications using an Olympus FV-1000 confocal/MPE laser-scanning microscope.

### Cell cycle analysis

Flow cytometry was used to assess whether chemical treatments arrested Raw 264.7 cells at any stage of the cell cycle. At the time of analysis, the cells were centrifuged, washed in PBS, fixed with cold methanol for two hours at 4°C and stained with a freshly made solution containing 0.1 mg/ml propidium iodide (PI) (Sigma-Aldrich) and 0.2 mg/ml ribonuclease A in PBS. All samples were incubated overnight in the dark at 4°C. Cell-cycle distribution was determined using a FACSCalibur flow cytometer (BD Biosciences) and data analyzed with FlowJo software on DNA instrument settings (linear FL2) on low.

### Macrophage polarization

Raw 264.7 cells were exposed to IFNγ (20 ng/ml, Peprotech) 24 h prior to treatment with Tuni, BFA or SKF96365 to generate the M1 inflammatory phenotype. Stimulated cells were removed from wells and incubated in anti-mouse BD Fc Block CD16/CD32 (BD Biosciences) for 30 min on ice in FACS buffer (PBS with 3% FBS), then surface-stained with PE-conjugated anti-iNOS (Cell Signaling Technology, D6B6S), anti-arginase-1 (Cell Signaling Technology, D43EM), anti-CD80 (Abcam, ab134120) and anti-CD206 (Abcam, ab64693) antibodies for 15 min at 4°C. Cells were washed three times with FACS buffer, then fixed with paraformaldehyde (4%) for 40 min at 4°C and washed three times in PBS. For intracellular staining, cells were permeabilized by adding ice-cold 100% methanol slowly to the cold cells with gentle vortexing, to a final concentration of 90% methanol. Cells were then incubated for 30 min at 4°C prior to staining with anti-arginase-1 primary antibody for 1 h at 4°C, cells were then washed three times by means of centrifugation at 400 ***g*** for 5 min with FACS buffer, and then incubated in the dark with FITC- and PE-labeled secondary antibody [FITC-conjugated goat anti-rabbit-IgG (Abcam, ab6717) and PE-conjugated goat anti-rabbit-IgG (Abcam, ab97024), both used at 1:50 dilution] 30 min at 4°C. Cells were washed three times by means of centrifugation at 400 ***g*** for 5 min, then analyzed using a BD LSR II flow cytometer (BD Biosciences) and FlowJo software version 9.0.

### Phagocytosis assay

After treatment, Raw 264.7 cells were assessed for phagocytic activity using the Phagocytosis Assay Kit (Cayman Chemical). Cells were incubated with latex beads with rabbit IgG–FITC conjugates (1:100) for 4 h followed by a 15-min incubation with Hoechst 33258 (Thermo Fisher) to quench non-phagocytosed bead fluorescence. We used LPS (100 ng/ml, Sigma-Aldrich) as a positive control, cells were tested following incubation for 3 h after treatments. Thereafter, the slides were washed, and coverslips mounted using VECTASHIELD HardSet Mounting Media with DAPI (Vector Laboratories, Inc.). Five images were acquired at 63× magnifications from each well using an Olympus FV-1000 confocal/MPE laser-scanning microscope. For the flow cytometry part of the assay, after incubation cells were washed and analyzed using a BD LSR II flow cytometer. The phagocytic index was calculated using the formula: phagocytic index=[(total number of engulfed cells/total number of counted macrophages)×(number of macrophages containing engulfed cells/total number of counted macrophages)]×100.

### Statistical analysis

Data analysis was performed using a one-way ANOVA or Student’s *t*-test on GraphPad Prism 8.0. Ca^2+^ measurement and electrophysiology were analyzed using Origin 9.0 software (OriginLab). Experimental values are expressed as means±s.d. *P*-values are represented as *P<0.05, **P<0.01 and ***P<0.001.
