# Supplementary material for: Loss of Ca2+ entry via Orai–TRPC1 induces ER stress, initiating immune activation in macrophages
Source: J Cell Sci. 2019 Dec 18;133(5):jcs237610. doi: 10.1242/jcs.237610 (PMC10682644; doi:10.1242/jcs.237610)
Supplement: Supplementary file 1 [file joces-133-237610_review_history.pdf]

Loss of Ca^{2+} entry via Orai-TRPC1 induces ER stress, initiating immune activation in macrophages

Viviane Nascimento Da Conceicao, Yuyang Sun, Emily K. Zboril, Jorge J. De la Chapa and Brij B. Singh
DOI: 10.1242/jcs.237610

Editor: Daniel Billadeau

Review timeline
Original submission: 5 August 2019
Editorial decision: 28 August 2019
First revision received: 27 September 2019
Accepted: 25 October 2019

Original submission

First decision letter

MS ID#: JOCES/2019/237610

MS TITLE: Loss of Ca2+ entry via Orai-TRPC1 induces ER stress that initiates immune activation in macrophage cells

AUTHORS: Viviane Nascimento Da Conceicao, Yuyang Sun, Emily Zboril, Jorge Chapa and Brij Singh
ARTICLE TYPE: Research Article

We have now reached a decision on the above manuscript.

To see the reviewers' reports and a copy of this decision letter, please go to: https://submit-jcs.biologists.org and click on the 'Manuscripts with Decisions' queue in the Author Area. (Corresponding author only has access to reviews.)

As you will see, both reviewers raise a number of substantial criticisms that prevent me from accepting the paper at this stage. In particular, there is a need to more clearly define the effect of TM or BFA on macrophage polarization and demonstrate that this is not just a phenomena seen in RAW cells. Does this occur in primary mouse or human macrophages. Moreover, as pointed out by reviewer 2, many of the immunoblots require loading controls and should be quantified over multiple experiments. They suggest, however, that a revised version might prove acceptable, if you can address their concerns. If you think that you can deal satisfactorily with the criticisms on revision, I would be pleased to see a revised manuscript. We would then return it to the reviewers.

Please ensure that you clearly highlight all changes made in the revised manuscript. Please avoid using 'Tracked changes' in Word files as these are lost in PDF conversion.

I should be grateful if you would also provide a point-by-point response detailing how you have dealt with the points raised by the reviewers in the 'Response to Reviewers' box. Please attend to all of the reviewers' comments. If you do not agree with any of their criticisms or suggestions please explain clearly why this is so.

© 2019. Published by The Company of Biologists under the terms of the Creative Commons Attribution License (https://creativecommons.org/licenses/by/4.0/).

Reviewer 1

Advance summary and potential significance to field

The report by Nascimento Da Conceicao et al describes a new role for Orai1, TRPC1 and STIM1 calcium channels in regulating ER stress. The authors propose here that BFA or tunicamycine (TM), both ER stress inducers; decrease Ca2+ release from the ER and extracellular Ca2+ entry. This loss of Ca2+ flux is followed by 1) down regulation of calcium channels expression (Orai1, TRPC1, STIM1), 2) induction of ER stress proteins (CHOP, IRE1, PDI), cytokine production, autophagy and 3) polarization of raw cells into M1 macrophages. In addition, the use of SKF inhibitor, which blocks extracellular Ca2+ uptake by Orai1 and TRPC1, also increase ER stress and cytokine production. The authors suggest a new link between ER stress and Ca2+ inducing cytokine production and autophagy.

Comments for the author

I feel major revision of this manuscript should be addressed before publication in JCS. The main problem with the conclusion of the paper, based on previous studies showing that XPB1 (an IRE1-dependent transcription factor induced upon tunicamycine treatment) acts in synergy with TLRs stimulation for optimal cytokine production in bone marrow derived macrophages (Martinon F et al, 2010, NI), is the extremely high level of cytokine detected in raw supernatant upon ER stress induction or blockade of extracellular Ca2+ uptake using SKF inhibitor. In the work of Martinon et al and works of others, macrophages stimulated with tunicamycine (TM) alone secrete low level of cytokines. In addition, BFA blocks the transport of proteins from the golgi apparatus to the ER and is used to monitor the expression of intracellular but not extracellular cytokines. Is this high level of cytokine production upon TM, BFA or SKF stimulation specific to raw cells? Is it also true for bone marrow derived macrophages? Do Orai1, TRPC1 or STIM1 deficient cells secrete high level of cytokines at the steady state?
Other remarks:
- The increase or decrease expression of calcium channels or ER stress proteins rely on WB experiments. However, for many WB the loading control (actin) is missing, for example Figure 1E, a WB for actin running at 45 kDa is showed with a WB for CHOP running at the same size (same in Figure 1G, Figure 3A, Figure 3C, Figure 3E, Figure 3G, etc.). Did the authors perform acid stripping? Can the authors provide the full Blot? Protein expression varies a lot from one experiment to the other. For example, Figure 1A, in CTRL lane very low GRP94 expression is detected, in Figure 1B in CTRL lane good GRP94 expression is detected, why such variation? (Also true for PDI, CHOP).
- No expression of IRE1 is detected in BMDM, this is very surprising (Figure 1E).
- Figure 4E: WB for Orai1 should be shown in SKF treated cells.
- Figure 4G: % of cell survival, in control more than 1?
- Figure 5C: the staining for CHOP in SKF treated cells is weak. SKF is inducing CHOP expression in raw cells (Figure 4C).
- Figure 6G show an ELISA but the description in the text is for a WB.
- Figure 7A: the legend of the histograms is difficult to read
- Figure 7F: raw cells are unable to phagocyte?
- Figure 8A: no MW is mentioned. The bands seem to be cut. What is the expression of IRE1 and the other stress proteins in this experiment?
- Can the authors explained their protocol for overexpressing ORAi1 and STIM1 in Raw cells (not detailed in mat and methods)

Reviewer 2

Advance summary and potential significance to field

Manuscript: Loss of Ca2+ entry via Orai-TRPC1 induces ER stress that initiates immune activation in macrophage cells. This investigation found that loss of Ca2+ entry via Orai-TRPC1 induced endoplasmic reticulum (ER) stress and in inflammatory reactions. The data showed that, in macrophages, when Ca2+ entry is inhibited, it directly leads to the activation of ER stress and cell

© 2019. Published by The Company of Biologists under the terms of the Creative Commons Attribution License (https://creativecommons.org/licenses/by/4.0/).

death by the increased expression of CHOP (a multifunctional transcription factor in the ER stress response which plays a major role in promotion of cell death), GRP94 and activation of IRE1α, a known protein involved in the regulation of UPR (unfolded protein response).
Furthermore, the expression of pro-inflammatory cytokines was activated, such as TNFα, IL-6, and IL-1β that activates downstream mediators of inflammation. These findings are interesting, and the study can be further improved by addressing the following concerns.

Comments for the author

The chief concern is about the role in macrophage polarization, which is proposed but never carefully assessed. They do not test the markers of M1 (TNFa, …) and M2 (CD80 low and CD206 high) with caution, with several typical markers and examining with multiple approaches (images, western and facs). The autophagy observation is neither mechanistic nor deep enough, with only measuring LC3 by western, etc. There are no data in animals or other distinct macrophages or primary human cells (especially for the major data), how the data can fit in physiological and clinical settings?
Some specific points as below:
1. In figure 7D, the results of flow cytometry, please present a positive control to demonstrate that the differences are significant.
2. In figure 8A, the bands of western blot were cut off and a bar diagram should be shown to represent densitometer values of the western blot images.
3. The representation of significant differences in all of your bar diagrams are not clear, please clearly indicate them.
4. There are some mistakes in language, please check and revise them.

First revision

Author response to reviewers' comments

We thank the reviewers for their appreciative comments and for the careful review of our manuscript. The suggestions made by the reviewers were very helpful and we have performed additional experiments and edited the manuscript accordingly to address all the points raised by the reviewers. These revisions have improved the manuscript considerably and we again thank the reviewers for these insights. Our detailed response for the review is as follows:

Reviewer 1:
1. The main problem with the conclusion of the paper, based on previous studies showing that XPB1 (an IRE1-dependent transcription factor-induced upon tunicamycin treatment) acts in synergy with TLRs stimulation for optimal cytokine production in bone marrow-derived macrophages (Martinon F et al, 2010, NI), is the extremely high level of cytokine detected in raw supernatant upon ER stress induction or blockade of extracellular Ca2+ uptake using SKF inhibitor. In the work of Martinon et al and works of others, macrophages stimulated with tunicamycin (TM) alone secrete low levels of cytokines. In addition, BFA blocks the transport of proteins from the Golgi apparatus to the ER and is used to monitor the expression of intracellular but not extracellular cytokines. Is this high level of cytokine production upon TM, BFA or SKF stimulation specific to raw cells? Is it also true for bone marrow-derived macrophages? Do Orai1, TRPC1 or STIM1 deficient cells secrete high level of cytokines at the steady-state?
Response: We are sorry for not making this aspect clear, actually, our data is consistent with Martinon et al., the only difference was that our ELISA results were shown in pg/ml, whereas data in Martinon et al (2010, figure 4A) is shown in ng/ml, which is 1000 fold less and thus could have caused this confusion. In addition, these authors co-stimulated the cells with LPS and Tunicamycin which is different from our experiments as different mechanisms might be involved in ER stress induction or TLR mediated stimulation. We agree that LPS is a well-known inducer of cytokine production, however, ER stress has also been shown to induce similar immune activation that might be critical for many diseases including autoimmune diseases. We agree with the reviewer that SOCE plays a key role in the regulations of cytokines. It has been already established that extracellular

© 2019. Published by The Company of Biologists under the terms of the Creative Commons Attribution License (https://creativecommons.org/licenses/by/4.0/).

Ca2+ influx upon TLR stimulation is required for the activation of immune cells especially in macrophages that modulates cytokine production (Vaeth et al., 2015). In contrast, increased expression of pro-inflammatory cytokines during ER stress has also been observed, however, the mechanism is not clear. The data presented in this manuscript provide important clues and we have performed experiments to understand how ER stress could lead to cytokine production and immune activation. We showed that ER stressors decrease calcium signaling which was due to the downregulation of the expression of calcium channels (TRPC1, Orai1) and its regulator (STIM1). Loss of this vital function could induce cytokine release, along with inducing cell death in the innate immune cells. To really establish the link between calcium signaling and ER stress, we blocked the SOCE channels with SKF 96365, which also induced ER stress and showed corresponding immune activation. Cytokine analysis was performed in macrophage cells that were treated not only by Tuni and BFA but also with SKF, showing that ER stress induction, which was dependent on proper calcium signaling. Moreover, restoration of SOCE decreased the expression of pro-inflammatory cytokines, such as IL-6, IL-1β, and TNF-α and stimulated protection of macrophages. Thus, our results provide a mechanism that shows that calcium is not only essential for TLR mediated cytokine production, but the loss of this vital pathway could also stimulate the release of cytokines in innate immune cells, which is the main aspect of this study. Importantly, others have reported that ER stress inducers (tunicamycin or BFA) induce the release of cytokines (Kim et al., 2012, Mahadevan et al., 2011), which is consistent with our studies. The minor differences observed in the scale could be due to the different cell lines used, as well as the length of treatment. We have modified the figures and have included the data as ng/ml to make this consistent with previous publication as well as discuss the role of TLRs in the discussion section.

In addition, as suggested by the reviewer we performed additional experiments using primary macrophage cells. Importantly, primary cells also showed similar cytokines levels, which is consistent with previous works such as Shenderov et al (2014) and have included the data in the revised version of the manuscript (supplemental figure 2). In addition, we also used LPS as a [positive control that also showed an increase in the cytokine production in these cells. Overall, our studies show that in the presence of Tuni, BFA, or SKF, both Raw 264.7 and the bone marrow-derived macrophages (BMDM) lead to the downregulation of the SOCE channels and its regulators, which further induces cytokine production. The cytokine assays were performed on the cells under the same conditions as shown in our western blot experiments (figure 6B). To have further insight into this we have also silenced those genes (as suggested by reviewer 2) and have performed ELISA to establish that it is the loss of these calcium signaling molecules that induce cytokine production and secretion. We also agree with the reviewer that BFA is an inhibitor of protein trafficking in the endomembrane system of mammalian cells, however, previous studies have shown to increase cytokine release in the media (Kim et al, 2012). Although we cannot confirm if this increase in cytokine is either due to ER stress-mediated release or could be an outcome from the apoptosis of the innate immune cells due to the 12hr treatment with BFA, where the cells would experience membrane collapse. We thank the reviewer for this important insight and have added this caveat in the results and discussion section.

2. The increase or decrease expression of calcium channels or ER stress proteins rely on WB experiments. However, for many WB the loading control (actin) is missing, for example, Figure 1E, a WB for actin running at 45 kDa is showed with a WB for CHOP running at the same size (same in Figure 1G, Figure 3A, Figure 3C, Figure 3E, Figure 3G, etc.). Did the authors perform acid stripping?
Response: We sincerely apologize for this oversight and thank the reviewer for pointing that out. As correctly pointed out by the reviewer, we have included loading controls in all experiments (please see the revised manuscript where molecular weights are correctly labeled for actin blots). We do apologize for the mistake where we accidentally showed that CHOP was the same size as Actin in figure 1E, which is obviously not correct as the molecular weight of CHOP is 27 kDa and Actin has an MW of 45kda, thus have not used acid stripping for the loading control. We have corrected this honest mistake in the revised manuscript and sincerely appreciate the careful review of our manuscript.

3. Can the authors provide the full Blot? Protein expression varies a lot from one experiment to the other. For example, Figure 1A, in CTRL lane very low GRP94 expression is detected, in Figure 1B in CTRL lane good GRP94 expression is detected, why such variation? (Also true for PDI, CHOP).
Response: We thank the reviewer for bringing this to our attention and we do not fully agree that protein loading is an issue as we run the same samples against loading control actin each time to

© 2019. Published by The Company of Biologists under the terms of the Creative Commons Attribution License (https://creativecommons.org/licenses/by/4.0/).

confirm the equal loading of samples in each lane. Similarly, each experiment is performed with (either Tuni, BFA or SKF) or without (Control) treatment and we have compared them from individual replicates. This is the reason that we have provided densitometry for all blots that are more consistent with the western blots itself as they are replicates of 3-4 individual experiments and have the desired statistics. We agree that there are some variations between different blots, which is not due to the loading control per se, but rather due to the different exposure times of the western blots. Thus, we have provided new western blots that truly represent the combined data which has rectified this problem.

4. No expression of IRE1 is detected in BMDM, this is very surprising (Figure 1E).
Response: As clarified above we have provided new western blots for figure 1E that represent the data appropriately and agree that in the previous manuscript some of the blots were not consistent.

5. Figure 4E: WB for Orai1 should be shown in SKF treated cells.
Response: As suggested by the reviewer, we have included the WB for Orai-1 in our SKF treatment (please see figure 4E in the reviewed manuscript).

6. Figure 4G: % of cell survival, in control of more than 1?
Response: We again thank for the careful review of our manuscript. The cell viability was expressed as a percentage of the control conditions, we apologize that we did not normalize our calculations, to take control as 1 or 100%. However, to make it clear, we have now normalized the data and have corrected this in the revised manuscript.

7. Figure 5C: the staining for CHOP in SKF treated cells is weak. SKF is inducing CHOP expression in raw cells (Figure 4C). Change figure on microscope or change blot
Response: As suggested by the reviewer, we have included a new figure for CHOP as well as have also included the expression of beclin1 in these cells in the revised manuscript.

8. Figure 6G shows an ELISA but the description in the text is for a WB.
Response: We thank the reviewer for carefully reading our manuscript. We do apologize for the oversight and have corrected this in the revised manuscript.

9. Figure 7A: the legend of the histograms is difficult to read
Response: Legends fonts have been increased to make them easier to read as suggested.

10. Figure 7F: raw cells are unable to phagocyte?
Response: We do not fully agree with the reviewer’s statement, as shown in the figure healthy macrophages (control) showed very little phagocytic index, which is consistent with the reviewer's point that raw cells in control conditions do not show phagocytosis. However, when compared with the cells under ER stress, phagocytosis was clearly established, which is consistent as it has been shown before that phagocytosis of pathogens by macrophages is an important mechanism of defense and initiates the immune response (Kim et al, 2018). We also used LPS to confirm that indeed these cells are able to phagocytosis, indeed M1 phenotype was observed upon the addition of ER stressors that is able to phagocytosis. We demonstrated here that under ER stress, the macrophages are active and increased phagocytosis level was observed under Tuni, BFA and SKF treatments.

11. Figure 8A: no MW is mentioned. The bands seem to be cut. What is the expression of IRE1 and the other stress proteins in this experiment?
Response: We thank the reviewer for pointing this editing mistake, we have provided the MW of the proteins shown in figure 8A. We also would like to state that we only used GRP94 and CHOP as a marker for ER stress during the overexpressed experiments rather than showing the same blots again. We made that decision since CHOP is a well-established transcription factor activated only during ER stress and it has been determined by several studies that GRP94 is only upregulated in response to ER stress and its role in cellular immune response has been studied intensely. Our overall goal here was to establish if restoration of Orai1 is sufficient to counteract the effect of Er stressors and have performed additional experiments as shown in the figure to make our point.

© 2019. Published by The Company of Biologists under the terms of the Creative Commons Attribution License (https://creativecommons.org/licenses/by/4.0/).

12. Can the authors explain their protocol for overexpressing ORAi1 and STIM1 in Raw cells (not detailed in mat and methods)
Response: We apologize for not detailing our overexpression protocol and have corrected this in the Materials and Methods section of the revised manuscript.

Reviewer 2:
1. The chief concern is about the role in macrophage polarization, which is proposed but never carefully assessed. They do not test the markers of M1 (TNFa,…) and M2 (CD80 low and CD206 high) with caution, with several typical markers and examining with multiple approaches (images, western and facs). The autophagy observation is neither mechanistic nor no deep enough, with only measuring LC3 by western, etc. There are no data in animals or other distinct macrophages or primary human cells (especially for the major data), how the data can fit in physiological and clinical settings?
Response: We thank the reviewer for his/her insight and would like to point it out that, in our flow cytometer experiments for the macrophage polarization, we use iNOS and Arginase 1, which are the classic markers for the M1 and M2 macrophages (Figure 7A). We have also complemented our findings with western blot for those markers. We also showed the secretion of pro-inflammatory cytokines for M1 macrophages, such as IL-6, IL-1β and TNFα (Figure 6A), demonstrating that's a clear indication of the high level of M1 macrophage present in our experiments (due to ER stress activation). In addition, we have also shown western blot of those cytokines and all these cytokines were upregulated by ER stress induction (Figure 6B) that reinforces our overall findings with different experimental strategies. We agree that CD80 low and CD206 high are also the markers for M2, and have used it to determine the M2 population in these conditions (supplemental figure 2B). We agree that we have not used primary human cells, but we have shown the ER stress data in primary mouse macrophage cells in figures 1 and figures 3, not only using western blotting but also with whole-cell patch recording, that shows ER stress induction as well as loss of calcium signaling when the cells are treated with Tuni and BFA. We also do not fully agree that the autophagy observed is not deep enough. Our data clearly showed that prolonged ER stress induction leads to apoptosis, which we have measured using different assays and autophagy has been shown to be the regulators of apoptosis where it could promote cell death as observed in these conditions. Loss of calcium signaling (mainly in the ER) has been suggested to induce ER stress in other cells, as well as ER stress has been a well-known activator for immune cells; however, the ion channels as well as if the similar phenomenon is present in macrophage cells is not clear. Here we provide the evidence that indeed SOCE is essential for the induction of ER stress and loss of SOCE via the Orai1 channel is essential for this pathway. This is physiologically relevant, as restoration of its expression restored calcium signaling as well as decreased ER-stress induced immune activation, which is observed in several autoimmune diseases. We have discussed this further in the discussion section of the revised manuscript.

2. In figure 7D, the results of flow cytometry, please present a positive control to demonstrate that the differences are significant.
Response: We thank the reviewer for this excellent suggestion and have included LPS to the cells as positive control and analyzed them by flow cytometer to show positive control as suggested (data provided in Figure 7D). In addition we also used LPS to look at cytokine release in these conditions, which has also been included in the revised manuscript.

3. In figure 8A, the bands of western blot were cut off and a bar diagram should be shown to represent densitometer values of the western blot images.
Response: We again thank for this excellent suggestion and have now included the densitometry values of the blots (supplemental figure 3) and have also provided new images of the western blots.

4. The representation of the significant difference in all your bar diagrams are not clear, please clearly indicate them.
Response: As suggested we have adjusted the representation of the bar graphs, consequently making the statistic differences in each condition more clear.

© 2019. Published by The Company of Biologists under the terms of the Creative Commons Attribution License (https://creativecommons.org/licenses/by/4.0/).

5. There are some mistakes in language, please check and revise them.
Response: We have gone through the manuscript carefully and have corrected the language or grammatical errors in the revised manuscript.

Second decision letter

MS ID#: JOCES/2019/237610

MS TITLE: Loss of Ca2+ entry via Orai-TRPC1 induces ER stress that initiates immune activation in macrophage cells

AUTHORS: Viviane Nascimento Da Conceicao, Yuyang Sun, Emily Zboril, Jorge Chapa and Brij Singh
ARTICLE TYPE: Research Article

I am happy to tell you that your manuscript has been accepted for publication in Journal of Cell Science, pending standard ethics checks.

Reviewer 1

Advance summary and potential significance to field

Role of calium sensors/pump in ER stress in Raw macrophages.

Comments for the author

I have carefully re-reviewed the paper by Nascimento Da Conceicao and I have some concerns:
point 1: there is still very high level of cytokine detected in raw supernatant upon ER stress induction only (not together with TLR stimulation) and I don't really understand their reply (pg versus ng). In both versions of the paper the level of cytokine secretion is very high around 1500 pg to 7000 pg/ml (1st version of the manuscript) and 2 to 7 ng/ml (2nd version of the manuscript).
point 2: there is still actin loading control missing for many western blots experiments (Figure 3A, C, E; Figure 4E etc...)
Supplemental Figure 2A: the cells are stimulated with what?, Supplemental Figure 4B: at the steady state raw cells are producing 0,5 to 1 ng/ml of cytokine and silencing calcium sensors/channels such as ORA1, STIM1 or TRPC1 increases the level of cytokine production to 2-4ng/ml. We tested this in STIM1 and ORA1 deficient mice 2 years ago and never saw this at the steady state.

Reviewer 2

Advance summary and potential significance to field

Authors have responded reasonably to my comments.

Comments for the author

Authors have responded reasonably to my comments.

© 2019. Published by The Company of Biologists under the terms of the Creative Commons Attribution License (https://creativecommons.org/licenses/by/4.0/).
